# Jugend und Alkohol: Eine Bestandsaufnahme mit Blick auf die Situation in Österreich

**DOI:** 10.1007/s40211-020-00365-7

**Published:** 2020-10-28

**Authors:** Katrin Skala

**Affiliations:** Universitätsklinik für Kinder- und Jugendpsychiatrie, Medizinische Universität Wien, Spitalgasse 23, 1090 Wien, Österreich

**Keywords:** Alkohol, Jugendliche, Österreich, Alcohol, Adolescents, Austria

## Abstract

Im vorliegenden Artikel wird auf die Rolle von Alkohol in unserer Gesellschaft sowie auf dessen Bedeutung für Jugendliche eingegangen. Spezifika der Wechselwirkung von Alkohol und dem adoleszenten Gehirn werden erläutert und die epidemiologische Entwicklung des Alkoholkonsums bei österreichischen Minderjährigen beschrieben. Es werden darüber hinaus relevanteRisiken für problematische Konsummuster und mögliche Wege zur Prävention erörtert.

## Summary

The present paper deals with the role of alcohol in our society and its importance for young people. Specifics of the interaction of alcohol and the adolescent brain are illustrated and the epidemiological development of alcohol consumption in Austrian minors is being described. Significant risks for problematic consumption patterns and possible ways of prevention are also discussed.

## Einleitung

Alkohol ist in unseren Breiten historisch tradiert die Kulturdroge schlechthin. Bereits im alten Rom und Griechenland gab es mit Bacchus und Dionysos Gottheiten des Weines und des Rausches [1] und Odysseus verwendete Alkohol sogar gleichsam als Waffe, indem er Polyphem gezielt betrunken machte, um ihn blenden zu können [2]. Auch in der Bibel wurde festgehalten, dass „Der Wein des Menschen Herz erfreuen möge“ [3]. Heute ist Alkohol zur Gebrauchsdroge geworden, stellt einen festen Bestandteil gesellschaftlicher Bräuche und Gepflogenheiten dar und dient der Festigung oder Knüpfung sozialer Kontakte im Umfeld festlicher Anlässe. Keine andere psychotrope Substanz wird von einem derart großen Prozentteil der Bevölkerung ohne relevante psychische oder somatische Folgen konsumiert [4].

Während Alkoholabhängigkeit im Jugendalter ein relativ seltenes Phänomen darstellt, ist Alkoholkonsum unter Jugendlichen ist in vielen Ländern der Europäischen Region sowie auch in Nordamerika ein verbreitetes Phänomen. Während das Suchen und Überschreiten von Grenzen gleichsam zu den Entwicklungsaufgaben der Adoleszenz gehören, wird riskantes Trinkverhalten, gerade bei Adoleszenten, mit einer Vielzahl an nachteiligen Folgen für die psychische, soziale und physische Gesundheit in Zusammenhang gebracht. In diesem Spannungsfeld aus Autonomieentwicklung und Abgrenzung qua psychotroper Substanz und dem Risiko gravierender Kollateralschäden muss der Konsum von Alkohol im Jugendalter reflektiert werden.

## Gesetzliche Rahmenbedingungen

Das Jugendschutzgesetz, welches mit 2019 bundesweit vereinheitlicht wurde, sieht ein gesetzliches Schutzalter von 16 Jahren für den Erwerb sowie Konsum von Bier und Wein in der Öffentlichkeit vor. Das Schutzalter für den Erwerb, Besitz und Konsum von Spirituosen, welches zuvor auf Länderebene uneinheitlich war, wurde österreichweit einheitlich auf 18 Jahre festgelegt [5]. Es gilt also festzuhalten, dass Erwerb, Besitz und Konsum von Alkohol in jeglicher Form für unter 16-Jährige illegal und der Erwerb, Besitz und Konsum von Spirituosen generell für Minderjährige gesetzlich nicht gestattet sind [6].

## Gehirnveränderungen im Jugendalter

Das Gehirn ist im Jugendalter ausgeprägten Umbauprozessen unterworfen. Es kommt zu einer Eliminierung gewisser Synapsen, dem sogenannten synaptischen Pruning. Im Zuge dieses Prozesses, bei welchem selektiv und vorwiegend im Frontallappen Synapsen rückgebildet werden, gehen rund 3 % der grauen Substanz des Gehirns verloren. Dieser Vorgang folgt dem Grundsatz des „use it or loose it“, das heißt, dass brachliegende Synapsen abgebaut werden während aktive Verschaltungen konsolidiert werden [7]. Das Gehirn arbeitet also mittels Erfahrung daran, aus der Masse möglicher Schaltkreise eine sehr kleine Auswahl zu treffen Dies ist hochrelevant für Entwicklung und Funktion des reifen Gehirns und für Prozesse wie Lernen und Erinnern unabdingbar.

Parallel zu diesem Prozess finde verstärkt Myelinisierung, von Neuriten durch Gliazellen die statt. Diese elektrische Isolierung der Nerven, die für eine effizientere Neuronenfunktion sorgt wird erst in der dritten Dekade abgeschlossen. Während phylogenetisch ältere Gehirnstrukturen wie etwa die Amygdalae verhältnismäßig früh myelinisiert werden, findet dieser Vorgang erst im frühen Erwachsenenalter im Frontalhirn seinen Abschluss [7].

Diese Prozesse stellen das biologische Korrelat für die Tatsache dar, dass im Jugendalter in der Regel Impulsivität die Kontrolle überwiegt und machen es nachvollziehbar, dass Jugendliche im Verhältnis zu Erwachsenen hohe Reagibilität und hohe Risikobereitschaft bei gleichzeitig geringer Risikoabschätzung zeigen. Auch Reflexions- und Antizipationsfähigkeit sind noch verhältnismäßig gering ausgeprägt, was bei gleichzeitig vorhandener hoher Vulnerabilität des Gehirns eine erhöhte Gefährdung durch exogene Noxen evident macht.

## Das Belohnungssystem (Reward Circuit) im Jugendalter

Die cerebralen Ursachen der Suchtentwicklung finden sich im meso-limbischen Belohnungssystem. Dieses hat seinen Ursprung in der Area tegmentalis ventralis des Mittelhirns und ist Teil des limbischen Systems. Die dopaminergen Neurone des mesolimbischen Systems projizieren mit ihren Axonen vor allem zu Strukturen des Vorderhirns, wie dem Nucleus accumbens, Striatum ventrale, den Amygdalae, dem Hippocampus, dem Cortex entorhinalis und dem Gyrus cinguli. Insbesondere durch Innervation des Nucleus accumbens, einer Kernstruktur der Basalganglien, werden dessen Ein- und Ausgangssignale moduliert. Letztere führen zu Strukturen wie dem Hypothalamus, dem Septum und dem Pallidum ventrale. Dieses Netzwerk mediiert assoziatives und emotionales Lernen, positive Verstärkung und klassische Konditionierung und zeichnet darüber hinaus für die Entstehung der Emotion „Freude“ verantwortlich. Drogen wie Opioide, Alkohol oder Nikotin aber auch Sport, Schokolade und im Grunde jedwede andere Substanz oder Tätigkeit, welche Suchtpotential hat, wirken prädominant durch direkte der indirekte Dopaminausschüttung im mesolimbischen System. Adoleszente Gehirne reagieren auch hier intensiver, sie sind sensibler auf Belohnung, und zeigen ein stärker ausgeprägtes „sensation seeking“ und „novelty seeking“ [8].

## Risiken bei juvenilem Alkoholkonsum

Seit längerem ist bekannt, dass Alkohol, seine toxische Wirkung im Gehirn überwiegend über die Inhibition der Neurogenese vor allem im Bereich des Präfrontaler Kortex, des Limbicums und des Cerebellums ausübt [9]. Dies äußert sich klinisch vor allem im Sinne von mangelnder Entwicklung intellektueller Funktionen und mangelnder emotionaler Reifung. Auch im Jugendalter reagieren weibliche Gehirne sensibler auf die toxische Wirkung von Alkohol und zeigen beim Konsum vergleichbarer Dosen stärker Defizite bei bestimmten kognitiven Aufgaben als die Gehirne ihrer männlichen Kollegen [10].

Es scheint, dass das Alters, in welchem regelmäßiger Alkoholkonsum initiiert wird, ausschlaggebend für die Art und das Ausmaß der Folgeerscheinungen ist. Jugendliche mit frühem Konsumbeginn, also solche, die bereits im Alter von 12–14 Jahren regelmäßig Alkohol trinken, haben im Vergleich zu ihren abstinenten Altersgenossen einen höheren Baseline-Konsum und fast zehnmal so häufig klinisch relevante Intoxikationen. Sie habendarüber hinaus ein höheres Maß an „stressful life events“ und sind im Alter von 19 Jahren fast siebenmal so häufig alkoholabhängig wie andere [11]. Auf schulischer und sozialer Ebene fallen sie durch höhere Impulsivität, riskantes Sexualverhalten, schlechtere schulische und neurokognitive Leistung und affektive Veränderungen auf [12].

Aus der bislang begrenzten Datenlage extrapoliert, scheint es, dass die regelmäßige Exposition gegenüber großen Alkoholmengen während der frühen und mittleren Jugend am ehesten das Sozialverhalten, die Belohnungssensitivität und affektive Funktionen beeinflusst, die besonders stark von subkortikalen limbischen Bereichen abhängen. Expositionen im späteren Jugendalter dürfte sich eher negativ auf die kognitive Entwicklung auswirken, die mit der langsameren Entwicklung des präfrontalen Kortex synchron ist [13]. Wegen der neurotoxischen Wirkungen der wiederholten Rebound-Effekte ist bei vergleichbaren Mengen Rauschtrinken deutlich schädlicher als chronischer Konsum. Ersteres führt rascher und expliziter zu Schädigungen im Gehirn [14].

Der Vollständigkeit halber muss erwähnt werden, dass aus den genannten Studien nicht letztendlich ableitbar ist, ob die beschriebenen Schädigungen in den Gehirnen junger Trinkern die Folge von Alkoholmissbrauch sind oder ob es sich hierbei vielmehr um vorbestehende Anomalien handelt, die die Jugendlichen zum Alkoholmissbrauch prädisponieren.

## Epidemiologie

Die Ergebnisse von Studien zur Prävalenz und Auswirkungen sogennannter Alkoholkonsumstörungen bei Jugendlichen unterscheiden sich stark. Dies ist zum einen dadurch bedingt, dass häufig eine klare Definition der Diagnose fehlt, zum anderen erschwert die oft heterogene Beschreibung der Konsummengen die Vergleichbarkeit. In einem wissenschaftlichen Kontext ist es wäre wünschenswert, sich beim Vergleich des Alkoholkonsums auf die Menge reinen Alkohols zu verlassen. Die Menge an reinem Ethanol kann ungefähr als 40 g in 1000 ml eines 5 %igen Getränkes wie Bier, 95 g in 1000 ml eines 12 %igen wie etwa Wein oder 316 g in 1000 ml eines 40 %igen Getränkes wie Schnaps geschätzt werden [15]. In den meisten Studien zum Alkoholkonsum werden jedoch Begriffe wie „drink“, „standard drink“ oder „unit“ verwendet. Während eine „unit“ als die Menge eines alkoholischen Getränkes definiert ist, die 10 g reinen Alkohol enthält, schwankt die Menge, die einem „standard drink“ zugesprochen wird zwischen 6 und 20 g reinen Alkohols. Für den Begriff des „drinks“, welcher am häufigsten in Studien zu juvenilem Alkoholkonsum verwendet wird, gibt es überhaupt keine Definition. Diese nicht abgestimmte Verwendung dieser Begriffe und der untersuchten Populationen macht es ist sehr schwierig, verschiedene Studien zu vergleichen [16].

### Situation in Österreich

Ungeachtet oben genannter methodologischer Schwierigkeiten gibt es europaweit zwei repräsentative Erhebungen, welche in regelmäßigen Abständen das Substanzkonsumverhalten von Kindern und Jugendlichen untersuchen. Das *„European School Survey Project on Alcohol and Other Drugs“ (ESPAD) *ist die weltweit größte Schülerbefragung zum Konsum von legalen sowie illegalen psychoaktiven Substanzen. Sie wurde in Österreich nach Untersuchungen in den Jahren 2003, 2007 und 2015 im Jahr 2019 zum insgesamt vierten Mal durchgeführt. Die *„Health Behaviour in School-aged Children“-Studie (HBSC)* erhebt seit 1986 im vier-Jahres Rhythmus Daten zu Gesundheit und Gesundheitsverhalten von Schülerinnen und Schülern in 42 europäischen Ländern.

**Figure 1:**
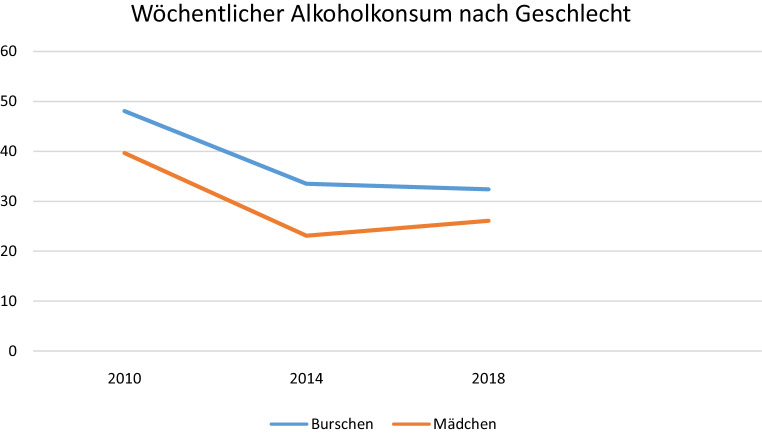

Diese Untersuchungen zeichnen den Alkoholkonsum österreichischer Jugendlicher betreffend ein weitgehend homogenes Bild. So dürfte der Einstieg in den Alkoholkonsum zwischen 13 und 15 Jahren zu liegen. Der wöchentliche Alkoholkonsum nimmt mit dem Alter deutlich zu und auch die wöchentlich konsumierte Menge an Alkohol steigt mit dem Alter deutlich an. In der Gruppe der 14–17-Jährigen haben 88 % schon Erfahrung mit Alkohol gemacht, bis zu zwei Drittel aller Befragten haben angegeben, zumindest einmal monatlich Alkohol zu konsumieren [17, 18]. Ein Fünftel der Jugendlichen berichtet davon, innerhalb des letzten Monats mindestens einmal berauscht gewesen zu sein und zwischen vier und sechs Prozent zeigten ein sogenanntes problematisches Konsummuster. Darunter ist ein Alkoholkonsum, welcher aufgrund der Frequenz (Konsum an 20 von 30 Tagen) und Konsummenge (mehr als 40 g reinen Alkohols bei Mädchen beziehungsweise 60 g bei Burschen) längerfristig mit einem relevanten Gesundheitsrisiko verbunden ist [17]. Konsummotive, die auf eine kompensatorische Funktion des Alkoholkonsums in Hinblick auf psychische oder psychosoziale Belastung hinweisen, wie der Konsum zur Behandlung depressiver oder ängstlicher Beschwerden werden hingegen nur von 2–10 % der Jugendlichen beschrieben [19]. Geschlechtsunterschiede im Konsumverhalten fallen bei Jugendlichen geringer aus als bei Erwachsenen, deutliche sind hingegen Unterschiede zwischen Schultypen sowie Unterschiede zwischen ländlichen und urbanen Regionen. SchülerInnen an polytechnischen Schulen und an Berufsschulen trinken deutlich häufiger und risikoreicher als jene aus weiterführenden Schulen, Jugendliche aus ländlichen Regionen häufiger und risikoreicher als solche aus urbanen Regionen ([17]; (s. auch Abb. 1, 2 und 3).

**Figure 2:**
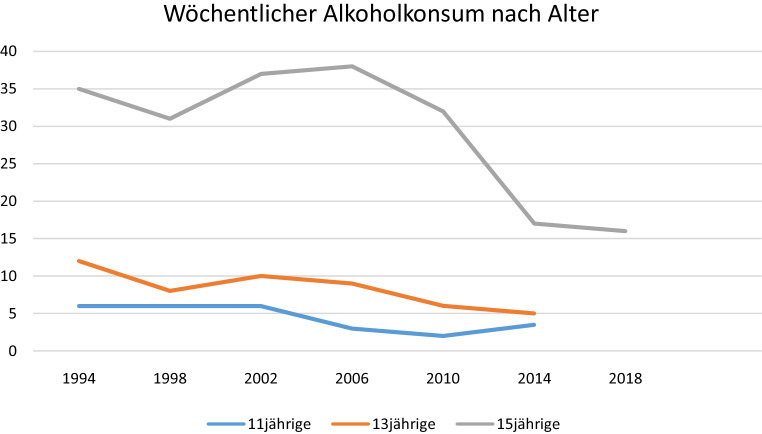

**Figure 3:**
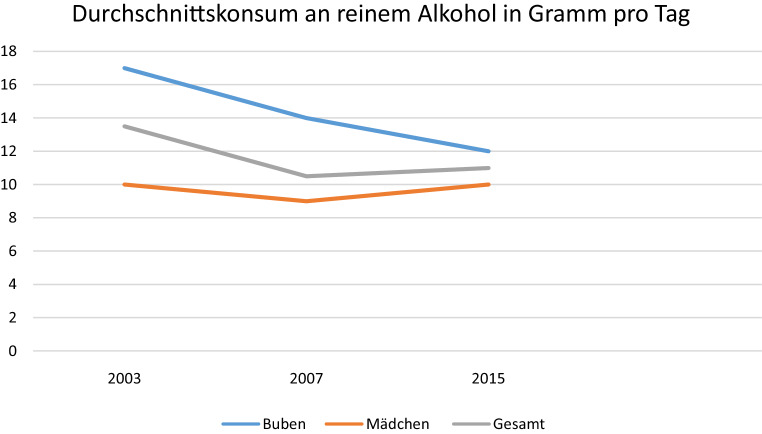

### Entwicklungen

Sowohl in der ESPAD-STUDIE als auch im Rahmen der HBSC-Erhebung wurde seit Beginn des Jahrhunderts ein Rückgang des Alkoholkonsums unter Jugendlichen festgestellt. So sanken etwa der Prozentsatz aktuell konsumierender Jugendlicher von 79 % auf 67 % und der tägliche Durchschnittskonsum von 14 auf 11 g. Parallel dazu stieg der Anteil lebenszeitabstinenter Jugendlicher von 4 % auf 12 % [17, 18]. Eine derartige rückläufige Entwicklung des Alkoholkonsums ist in vielen europäischen Ländern zu beobachten, sodass Österreich dennoch, sowohl Frequenz als auch Menge des Konsums betreffend, nach wie vor im Europäischen Spitzenfeld liegt [20].

### Binge, Rausch und Komasaufen

Ein wenig gesondert behandelt werden sollte das Thema des „Komasaufens“. Aus dem englischen Begriff des „binge drinking“ übertragen, stellt dieser Terminus die Bezeichnung für den Konsum von 60 g reinen Alkohols oder mehr für Männer beziehungsweise 40 g reinen Alkohols oder mehr für Frauen während eines „Trinkereignisses“ dar [21]. Konkret entspricht der Konsum von einem Liter Bier für Frauen und der Konsum von 1,5 l Bier für Männer an einem Trinkereignis bereits der Definition eines „binge drinking events“ und also des „Komasaufens“. Wiewohl es durchaus sinnvoll und wichtig ist, sich derartige Tatsachen vor Augen zu führen, ist es doch überraschend, dass zuletzt 68,7 % der 14-Jährigen und 78,4 % der 16-Jährigen angaben, ein derartiger Trinkereignis innerhalb des letzten Monats gehabt zu haben. Im Gegenzug dazu, gaben nur 33,6 % der 14-Jährigen und 41,9 % der 16-Jährigen an, in ihrem Leben einmal so betrunken gewesen zu sein, dass sie beim Gehen hin und her geschwankt sind, nicht mehr ordentlich sprechen konnten oder sich übergeben mussten [22].

Seit Mitte der 1990er-Jahre wurde eine starke Zunahme alkoholbedingter Spitalsaufnahmen von Jugendlichen in Österreich beobachtet. Diese gipfelt ungefähr Ende der Nullerjahre dieses Jahrhunderts. Während sich in den Folgejahren bei den unter 15-Jährigen wieder ein deutlicher Rückgang von Spitalsvorstellungen in Zusammenhang mit Alkoholkonsum zeigt, bleiben die Zahlen bei den über 15-Jährigen weitgehend stabil [16].

Dieser Anstieg scheint neben der Tatsache, dass das Thema „Jugend und Alkohol“ um die Jahrtausendwende zunehmend ins mediale Scheinwerferlicht rückte und die Bereitschaft der Ärzteschaft, derartige Fälle als solche zu diagnostizieren und zu dokumentieren, gestiegen ist, durch zwei weitere Faktoren begründet zu sein. Zum einen ist unter Jugendlichen eine allgemeine Tendenz zur Akzeleration und somit zur Ausübung „erwachsener“ Verhaltensweisen bereits in immer jüngerem Alter zu beobachten, zum anderen dürften sich zunehmend auch in Österreich, einem Land mit an sich „mediterranem“ und also moderatem aber kontinuierlichem Alkoholkonsum das nordeuropäische, exzessive Konsumverhalten ausbreiten [23]. Die Frage, ob es auch eine erhöhte Bereitschaft unter Jugendlichen gibt, intoxikierte Bekannte ins Krankenhaus einliefern zu lassen, muss hier unbeantwortet bleiben. Veritabel bedrohliche oder komatöse Zustände stellen bei, wegen Alkoholintoxikation aufgenommen, Jugendlichen jedoch eine seltene Ausnahme und keinesfalls die Regel dar [24].

## Risikofaktoren für juvenilen Alkoholkonsum

Antworten auf die Fragen, warum Jugendliche Alkohol konsumieren und warum sich bei manchen daraus ein missbräuchlicher Konsum beziehungsweise eine Abhängigkeit entwickelt, sind zentral für Prävention und Behandlung. Als Ursache für einen problematischen Umgang mit Alkohol wird übereinstimmend eine multifaktorielle Genese angenommen, das heißt, der Einfluss zahlreicher Faktoren beziehungsweise deren Wechselwirkung spielen hier eine Rolle. Unterschiedliche Entstehungsmodelle versuchen, die vielfältigen Interaktionen zu veranschaulichen.

### Persönliche und familiäre Faktoren

Die Tatsache, ein Kind suchtkranke Eltern zu sein, gefährdet in mehrfacher Hinsicht. Zum einen wird davon ausgegangen, dass es eine klare genetische Komponente für die Entwicklung von Suchterkrankungen gibt, zum andern stellt das Aufwachsen mit einem suchtkranken Elternteil eine erhebliche psychische Belastung dar. Es wird davon ausgegangen, dass in Österreich ungefähr zehn Prozent aller Kinder und Jugendlichen bis zum Alter von 18 Jahren mit der Alkoholabhängigkeit eines Elternteils oder beider Eltern konfrontiert sind [4]. Der häufige Aufenthalt in kontrollfreier Umgebung, mangelnder Rückhalt in der Familie und leichter Zugang zu Alkohol gelten als weitere Risikofaktoren [25]. An Persönlichkeitsmerkmalen stellt ein spezifisch ausgeprägtes Neugierverhalten („novelty seeking“ oder „sensation seeking“) einen prognostischen Faktor für den frühen den Konsum psychotroper Substanzen dar [26], auch unsichere Bindungsmuster sind klar als Risikofaktoren für Suchterkrankungen im allgemeinen und Alkoholabhängigkeit im speziellen erhöhen [27]. Häufig findet sich ausgeprägter juveniler Alkoholkonsum weiters co-morbid bei Jugendlichen mit Aufmerksamkeitsdefizit-Hyperaktivitäts-Syndrom, Angststörungen und depressiven Syndromen sowie bei Jugendlichen, welche in ihrer Geschichte frühe Traumatisierungen aufweisen [28].

### Frühe Gewöhnung an Alkohol

Je früher Alkoholkonsum initiiert wird, desto größer ist das Risiko von späterem Missbrauch und späterer Abhängigkeit [29, 30]. Auch wenn dieser Zusammenhang als valide gilt [31, 32], ist letztendlich doch unklar, ob es präexistente Gehirnveränderungen sind, die zu frühem Alkoholkonsum prädisponieren, oder ob es sich vielmehr um alkoholbedingte Veränderungen der Gehirnentwicklung handelt, welche die Wahrscheinlichkeit einer späteren Suchterkrankung erhöhen [12].

## Suchtprävention

Der Zugang zu alkoholkonsumierenden Jugendlichen steht in einem Spannungsfeld aus der Tatsache, dass das Übertreten von Grenzen gleichsam zu den Entwicklungsaufgaben Adoleszenter gehört und es klare Hinweise darauf gibt, dass isolierter Alkoholkonsum im mittleren und späteren Jugendalter ohne assoziierten psychischen Problemen keinerlei prädiktive Validität für negative Konsequenzen im weiteren Leben zeigt [33–35], und den etablierten Erkenntnisse zu altersbedingten neuronalen, verhaltensbezogenen und kognitiven Folgen von Alkohol-Exposition.

### Prohibition und strukturelle Prävention

Prohibition, also das Verbot einer Substanz oder Substanzgruppe stellt für Alkohol in unseren Breiten keine praktikable Lösung dar. Zum einen kann diese nur mit breiter sozialer Akzeptanz gelingen, zum anderen darf gerade bei Adoleszenten auch der Aspekt des erhöhten Reizes des Verbotenen nicht außer Acht gelassen werden. Strukturelle Prävention im Sinne von verstärkten vollständigen oder situativen Verboten, beschränkten Öffnungszeiten und erhöhten Preisen kann für Jugendliche bezüglich des Konsumbeginns jedoch präventiv wirken [36, 37]. Die Wirksamkeit der Schutznormen kann jedoch nur dann gewährleistet werden, wenn die Normen konsequent umgesetzt und Verstöße verlässlich und empfindlich sanktioniert werden. Der Einfluss gesetzlicher Beschränkungen auf den Konsum von Alkohol ist folglich in Österreich limitiert [16]. Jugendliche erachten es trotz etablierter strukturell präventiver Maßnahmen im Allgemeinen als einfach, Alkohol zu beschaffen und berichten in bis zu 70 % davon, Alkohol im häuslichen Umfeld, also von Eltern oder Geschwistern zu erhalten [38].

### Prävention

Suchtprävention in Zusammenhang mit Alkohol soll in erster Linie darauf abzielen, problematische oder gesundheitsschädliche Auswirkungen des Konsumverhaltens beziehungsweise die Entwicklung einer Abhängigkeitserkrankung zu verhindern [39]. Multiple Ansätze im Rahmen von des familiären [40, 41] oder schulischen [42, 43] Umfelds oder im Krankenhaussetting [44] durch Förderung der Lebenskompetenz und Wissensvermittlung über psychoaktive Substanzen und Suchtmechanismen präventiv zu arbeiten, zeigten sämtlich nur mäßige Erfolge. Als wirksamste Strategie der Alkoholprävention hat sich bis dato die Identifikation und das „case management“ gefährdeter Personen und Personengruppen herausgestellt [45].

## Conclusio

Zusammenfassend kann gesagt werden, dass Alkoholkonsum im Jugendalter ein ausnehmend weit verbreitetes Phänomen darstellt. Gehirnveränderungen im Jugendalter prädisponieren zu risikoreichem Konsum, das juvenile Gehirn reagiert aber gleichzeitig, zumindest bei jungen Adoleszenten, sensibler auf die toxische Wirkung des Alkohols. Sowohl Konsumfrequenz als auch konsumierte Menge an Alkohol sind in den letzten zehn Jahren in Österreich rückläufig und aktuell zeigen rund fünf Prozent aller Jugendlichen ein problematischen Konsumverhalten. Effiziente vorbeugende Maßnahmen zur Verhinderung gesundheitsschädlicher Auswirkungen und der Entwicklung einer Abhängigkeitserkrankung beinhalten in erster Linie die Identifikation und spezifische Betreuung gefährdetes Personen.
